# Evolutionary mechanisms underlying the diversification of nuclear factor of activated T cells across vertebrates

**DOI:** 10.1038/s41598-023-33751-6

**Published:** 2023-05-08

**Authors:** Maribet Gamboa, Noriko Kitamura, Kento Miura, Satoko Noda, Osamu Kaminuma

**Affiliations:** 1 Department of Disease Model, Research Institute for Radiation Biology and Medicine, Hiroshima University, Hiroshima, 734-8553 Japan; 2 Department of Ecology, Faculty of Sciences, Universidad Católica de la Santísima Concepción, 4090541 Concepción, Chile; 3 Neurovirology Project, Tokyo Metropolitan Institute of Medical Science, Tokyo, 156-8506 Japan; 4 Graduate School of Science and Engineering, Ibaraki University, Ibaraki, 310-8512 Japan

**Keywords:** Evolution, Immunology

## Abstract

The mechanisms of immunity linked to biological evolution are crucial for understanding animal morphogenesis, organogenesis, and biodiversity. The nuclear factor of activated T cells (NFAT) family consists of five members (NFATc1–c4, 5) with different functions in the immune system. However, the evolutionary dynamics of NFATs in vertebrates has not been explored. Herein, we investigated the origin and mechanisms underlying the diversification of NFATs by comparing the gene, transcript and protein sequences, and chromosome information. We defined an ancestral origin of NFATs during the bilaterian development, dated approximately 650 million years ago, where NFAT5 and NFATc1–c4 were derived independently. The conserved parallel evolution of NFATs in multiple species was probably attributed to their innate nature. Conversely, frequent gene duplications and chromosomal rearrangements in the recently evolved taxa have suggested their roles in the adaptive immune evolution. A significant correlation was observed between the chromosome rearrangements with gene duplications and the structural fixation changes in vertebrate NFATs, suggesting their role in NFAT diversification. Remarkably, a conserved gene structure around NFAT genes with vertebrate evolutionary-related breaking points indicated the inheritance of NFATs with their neighboring genes as a unit. The close relationship between NFAT diversification and vertebrate immune evolution was suggested.

## Introduction

Nuclear factor of activated T cells (NFAT) plays an important role in the immune system by regulating the transcription of multiple cytokines in T cells^1^. The NFAT protein family consists of five members, NFATc1–c4 and NFAT5, which share a DNA-binding region known as the Rel-homology domain (RHD)^1,2^. However, NFATc1–c4 (NFATcs) activation is regulated by a Ca^2+^/calmodulin-dependent serine/threonine phosphatase, calcineurin, which binds to the calcium regulatory domain (CRD) of NFATcs^3^, while the activation of NFAT5 occurs in response to osmotic stress^1^. NFATcs shuttle between the cytoplasm and nucleus in an intracellular Ca^2+^-dependent manner^2^, whereas NFAT5 is distributed throughout the cells^4^. Furthermore, differences in their expression in each cell type or organ, their structure, and contribution to each target gene results in the functional diversity of NFATs^2,5–7^. Owing to such diverse functions, structural abnormalities of NFATs potentially cause various diseases^8^. In addition to providing novel information in the quest to understand diseases, differences among NFATs are considered as targets of treatment regimens^9,10^. However, studies on the variation and evolution of NFATs and the mechanisms involved in their diversity are limited.

A remarkable association between NFAT and vertebrate evolution has been previously suggested. Three hypotheses have been proposed to explain the possible evolutionary origins and diversification of NFATs across vertebrates. First, the incidence of a recombination event in the RHD was hypothesized. Based on comparative studies of RHDs between fruit flies and five vertebrates (human, zebrafish, chicken, mouse, and hamster), it was hypothesized that the RHD in insects undergoes two independent recombination events, one with a regulatory region responsive to tonicity signals that generated NFAT5, and another with CRD that generated NFATcs^11^. Secondly, the incidence of an ancestral duplication event was hypothesized. The ancestral invertebrate-NFAT gene might have undergone mutations selected by natural selection, resulting in three duplication events. One duplication generated NFAT5, another generated NFATcs, and the duplication among NFATcs occurred before chordate evolution^12^. Thirdly, the incidence of a Ca^2+^ related vertebrate-specific recombination event was hypothesized. The recombination event that generated NFATcs, proposed in the first hypothesis, might be implicated in vertebrate organogenesis because the recombination of CRD possibly occurred only during vertebrate diversification^13^. All these hypotheses suggest the possible origin and diversification of NFATs from an ancestral invertebrate NFAT-like protein. However, questions regarding their evolutionary history among vertebrates, such as the timing of NFAT diversification events, drivers of evolutionary mechanisms in their diversification across vertebrates, and degree of conservation of NFATs among vertebrates remain unanswered.

Here, we address these questions by investigating the evolutionary history of NFATs and the possible evolutionary mechanisms associated with their structural and functional diversity across vertebrates. Gene duplication, alternative splicing, and chromosome rearrangement are three major evolutionary mechanisms that can cause functional and molecular variation by increasing gene diversification^14,15^. Although these mechanisms are important drivers of the evolution of transcription factors^16^, little is known about their role in NFAT evolution. Following the investigation of the phylogenetic relationships of NFATs across vertebrates that elucidated ancient diversification events, we focused on the three mechanisms of vertebrate NFAT evolution. Upon comparative analyses of protein and transcript sequences with the chromosome information that indicated the relationship between chromosomal evolution and gene duplication in NFATs, we further addressed the molecular evolutionary driver of NFATs. Lastly, in addition to evaluating the positive selection signal in several vertebrate taxa, we identified an outstanding gene order conservation in NFATs and the neighboring genes across vertebrate chromosomes.

## Results and discussion

### Ancestral and evolutionary conservation of NFATs

#### Ancestral NFATs date around bilaterians

**Figure 1:**
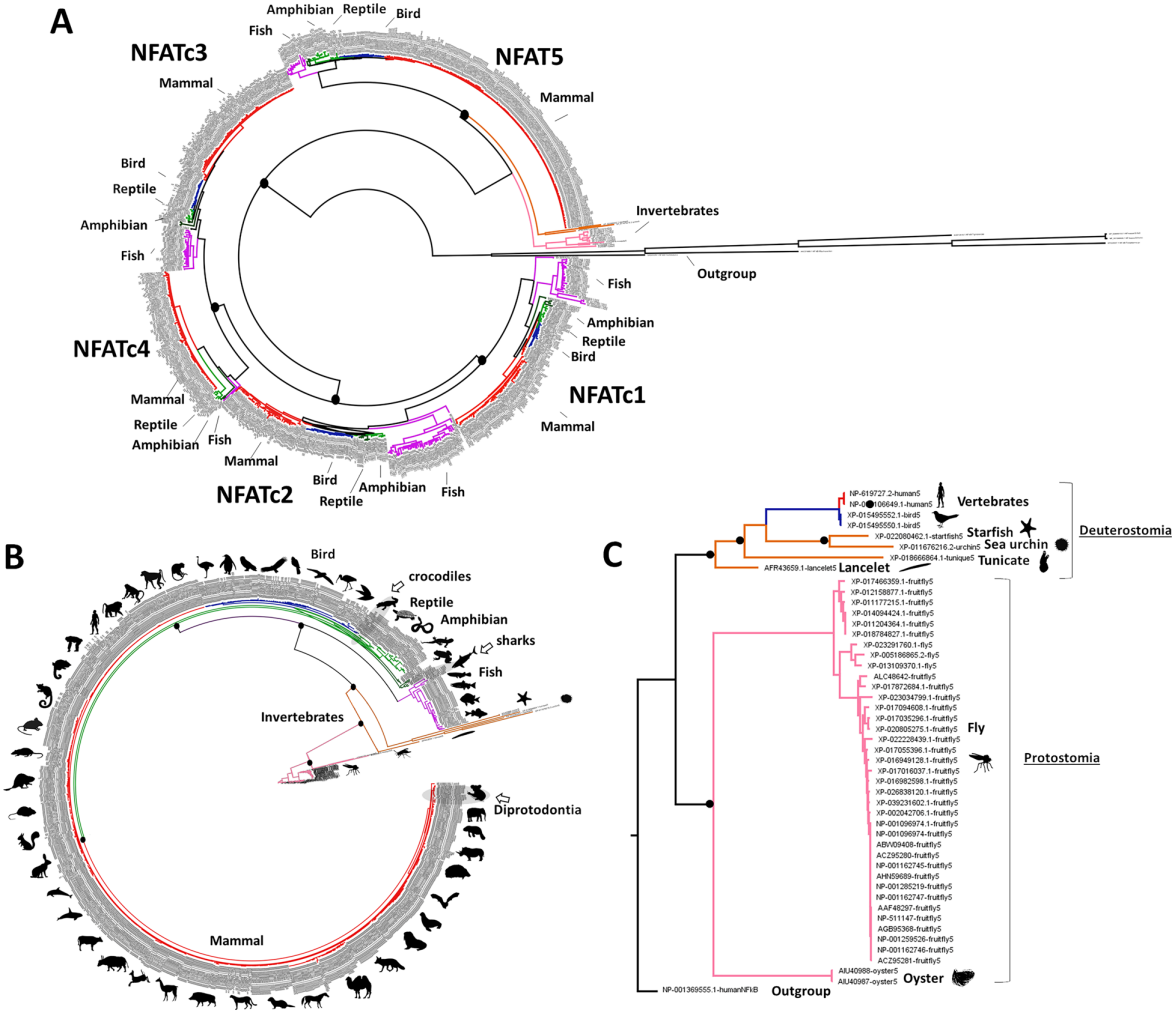
Evolutionary dynamics of NFATs across vertebrates. (**A**) A phylogenetic tree representing the evolutionary relationship among NFATs across vertebrate and invertebrate taxa. (**B**) NFAT5 phylogenetic tree is expanded to illustrate its detailed evolutionary divergence across vertebrates and invertebrates. (**C**) A phylogenetic tree representing the basal region of NFAT5, including two clades of Nephrozoa (Deuterostomia and Protostomia). Black dots on the major nodes represent maximum likelihood bootstrap (> 97) and Bayesian posterior probability (> 0.98) support values. Representative species in major vertebrate branches are illustrated as black silhouette images (http://phylopic.org/). Red, blue, light green, dark green, purple, orange, and pink branches correspond to mammal, bird, reptile, amphibian, fish, Deuterostomia, and Protostomia, respectively.

We explored the evolutionary dynamics of NFAT proteins, over a well-supported vertebrate and invertebrate phylogeny, reconstructed from 372 species (Table S1). Our phylogenetic reconstruction strongly indicated two independent origins for NFAT5 and NFATcs (Fig. 1A). NFAT5 first diverged independently of its ancestor, while another independent diversification led to the origin of NFATcs. These findings partly agree with those of previous reports suggesting the diversification of NFAT5 and NFATcs from an ancestral invertebrate NFAT-like^11,12^. Consistent with the identification of immune-related genes in the invertebrates, which suggests their role in the ancient immune system^12,17,18^, our results displayed invertebrate taxa as a basal group for NFAT5 phylogeny (Fig. 1B). However, the origins of NFAT5 and NFATcs did not fit the invertebrate NFAT-like hypothesis, suggesting the appearance of an NFAT-like protein before the development of invertebrates. The ancestral NFAT-like was most likely to date around the diversification of bilaterians during the Ediacaran geological period of Nephrozoa formation approximately 650 million years ago (Mya)^19^. Nephrozoa are a major diversification clade of bilaterians divided into two groups: Deuterostomia (vertebrates, starfish, and tunicates) and Protostomia (mollusks and arthropods)^20^. We identified NFAT5 in arthropods (flies and fruit flies), echinoderms (starfish), mollusks, deuterostomes (urchins), and Chordata ancestors (as tunicates and lancelets). NFAT5 formed two clades corresponding to the Nephrozoa group (Fig. 1C), suggesting that the origin of NFATs dates back to the period of Nephrozoa development.

Based on the independent origins of NFATcs, we proposed additional hypotheses. (1) Origin of Recombination. NFAT5 and a possible Nephrozoa NFAT-like protein remained together until approximately 550 Mya during the vertebrate formation^21^. The RHD of Nephrozoa NFAT-like might suffer a recombination event with a Ca^2+^-regulatory region that led to the origin of NFATcs, as proposed in the RHD recombination hypothesis^11^. This is consistent with the development of a Ca^2+^-dependent regulatory system as a vertebrate-specific event^13^. (2) Transposon element capture origin. Transposon elements are DNA sequences capable of changing their positions within a genome^22^. Many transcription factors have been proposed to originate from transposons^23^. An ancestral gain of a transposon-derived DNA-binding region, mostly originating from virus genomes, plays a pivotal role in vertebrate history^24^. Interestingly, several viral proteins contain conserved NFATc motifs^25^, suggesting the participation of transposable elements in NFATc evolution. Recombination events between Nephrozoa NFAT-like and transposable elements may contribute to NFATc diversification. (3) de novo origin. This hypothesis has been proposed for many transcription factors^26^. NFATcs contain a poorly conserved RHD^27,28^ that shows 18–20% similarity with NFAT5 and other RHD-containing proteins, such as nuclear factor kappa B (NF-κB)^27^. There were no detectable similarities between NFATcs and NFAT5 outside the RHD^11^, suggesting that their evolutionary affinities were obscured by rapid evolution. Substantial molecular diversity is also observed in other transcriptional factors, such as homeobox and helix-turn-helix^29^. Therefore, it is still possible that NFATcs were de novo generated during vertebrate evolution. Although our results suggested the existence of an ancestral NFAT-like protein, they did not show an obvious divergence pattern. Comparative analysis of RHD-containing proteins in a large number of organisms may contribute to reconstructing the evolutionary history of NFATs.

Within the clade of NFATcs, we found a bifurcation forming two clades: one clade containing NFATc1 and NFATc2, and another containing NFATc3 and NFATc4 (Fig. 1A), which were consistent with a previous report^12^. This diversification may be caused by a duplication event^12^ or by a vertebrate-specific translocation event^13^. However, these two clades were related to differences within the CRD^10^. CRD consists of three calcineurin-binding regions (CNBRs) with amino acid differences^9^. The amino acid sequences SPRIEIT and ITSISP in the N-terminal CNBR1 are conserved in NFATc1/NFATc2 and NFATc3/NFATc4, respectively, suggesting a link between CNBR1 and NFATc diversification. Functional differences among NFATcs have been reported^9^, hence, a detailed comparative analysis of amino acid differences across vertebrates is required for further interpretation of NFATc diversity.

#### Conserved NFATs across vertebrates

A conserved evolution within each NFAT was detected across vertebrates (Fig. 1B, Fig. S1). The gene trees were substantially similar to the species tree proposed by paleontological evidence^21^. For all NFATs, the Diprotodontia order of marsupials (platypus, opossum, kangaroo, and koala) showed a dichotomy from those in mammals, whereas the order Crocodilia (crocodilians and alligators) was closely related to that of birds. Similarly, the divergence between cartilaginous (sharks) and jawless fish lineages was in agreement with a whole-genome duplication event that occurred in cartilaginous fish during immune gene evolution^30^. Previous studies have reported the involvement of NFATs in evolutionarily ancient immune systems, wherein they served as components of effective, conserved defense strategies^13,17^; this suggests that the evolutionary conservation of NFAT may be associated with its innate immune nature.

Regardless of the similarities in the gene and species trees, NFATc4 was not found in birds (80 species, Fig. S1). Gene loss is considered when homologies are absent within the clade but are present in the closest sister lineage^31^. NFATc4 was identified in closely related taxa, such as reptiles, suggesting its loss during the diversification of birds from the other vertebrates. NFATc4 is associated with several immune functions, including the regulation of the Toll-like receptor^32^ and immunoglobulin E genes^33^ in the vertebrates. They have evolved into genes with specific functions in the birds^34,35^. The frequent loss of innate immune genes in birds, including that of NFATc4, may be related to the coevolutionary history of pathogens and the corresponding rapid defense system^36,37^.

Within mammals, a supported node bifurcation (bootstrap > 92/0.9 ML/Bayesian support) was found among great apes (Hominidae family: humans, gorillas, orangutan, and chimpanzees) for NFATcs (Fig. S1, Supplementary file 1). The resulting two isoforms correspond to alternative splicing isoforms as previously described^38^. NFATc isoforms have been reported to be associated with tissue specificity and/or disease in humans^39,40^. Here, we identified alternative splicing isoforms in the rest of the species belonging to the great ape lineage. Further studies on the evolutionary history of these isoforms, associated with great ape development, may be useful to understand the evolutionary nature of NFATcs in the recently evolved vertebrate taxa.

### Mechanisms underlying the diversification of NFATs across vertebrates

#### Gene duplication events mostly occurred during recent vertebrate diversification

**Figure 2:**
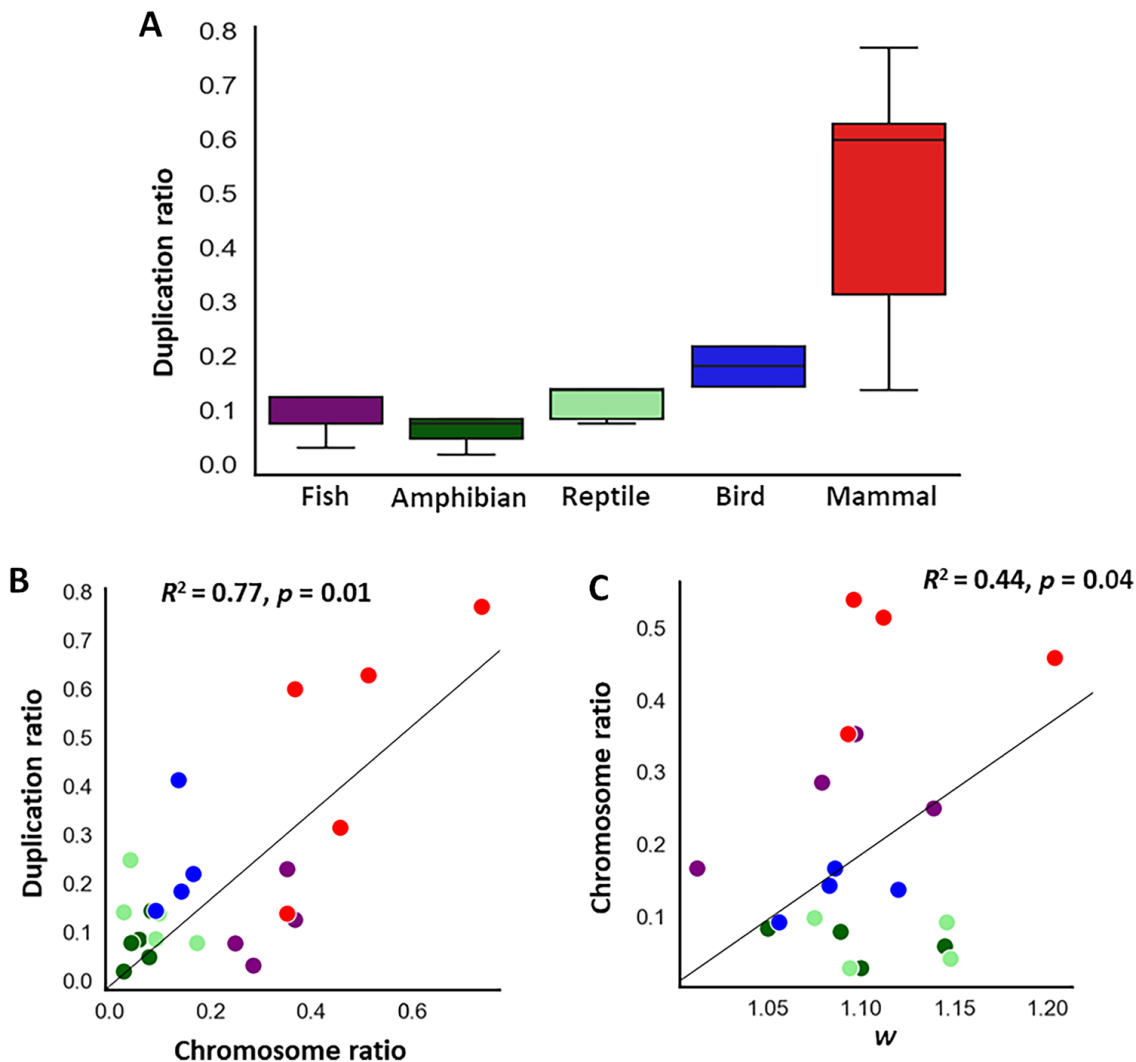
Mechanisms underlying the NFAT diversification across vertebrates. (**A**) Duplication ratio events observed in five major vertebrate taxa. The box and whisker plot of duplication ratio distribution in vertebrate NFATs is shown. The length of the box represents the interquartile range (IQR), the horizontal line in the box represents the median. The whiskers represent the 1.5 IQR of the 25th quartile or 1.5 IQR of the 75th quartile. (**B**) Relationship between chromosome and duplication ratios. The scatter plot of chromosome and duplication ratios evaluated in individual NFATs is shown with the regression line, coefficient of determination (*R*^2^) and *p*-value. (**C**) Relationship between chromosome ratio and fixation probability in positively selected NFATs. The scatter plot of chromosome ratio and fixation probability as the ratio (*w*) of nonsynonymous and synonymous substitutions in NFATc1-3, and NFAT5 is shown with the regression line, *R*^2^ and *p*-value. Red, blue, light green, dark green and purple correspond to mammal, bird, reptile, amphibian, and fish, respectively.

Gene duplication events are one of the candidate forces driving the evolution of NFAT^13,41^. To quantify the effects of duplication events among NFATs, we calculated the duplication ratio in vertebrate taxa using orthologous analysis. Among the 790 duplication events identified, the most frequent were observed in NFATc1, and each vertebrate taxon displayed an independent duplication pattern (Table S2). Duplicated events in all NFATs revealed a progressively ascending distribution in the recently evolved vertebrates (Fig. 2A). Even when analyzed in individual NFATs, the duplication ratio significantly correlated with the evolutionary time of vertebrate divergence (*R*^2^ = 0.71, *p* = 0.01, Fig. S2). Duplication events have been frequently observed during the recent evolutionary vertebrate diversification^42^ and are associated with speciation-related new gene creation^43^. Furthermore, evolutionary duplication events have been implicated in the adaptive immune system^44^, where NFATs play pivotal roles^45^. Altogether, the contribution of NFAT gene duplication events to the evolution of vertebrate immune systems was strongly suggested.

#### Low alternative splicing across vertebrates

The possible involvement of alternative splicing, which enables the production of multiple proteins from a single gene, in NFAT diversification has been reported, particularly, in human and mouse species^2,38^. To elucidate the origins of divergent NFAT isoforms, we examined NFAT-specific splicing events among vertebrates. Among the few alternative splicing isoforms identified in NFATs (Table S3), those in NFATc1 were the most frequent (46% of total gene splicing). This finding is partly consistent with the results of a previous report, which demonstrated that duplication events in NFATc1 are relatively frequent, owing to its stronger splicing tendency^46^.

The splicing pattern of NFAT was almost absent in older taxa, such as fish, whereas mammals showed significantly higher splicing ratios (Table S3), suggesting that the beginning of most splicing events occurred in recent taxa. A rapid divergence of gene expression has been identified in mammals^47^, mainly due to species divergence^48^, which suggests an unessential contribution of alternative splicing events to vertebrate NFAT evolution. Additional analyses using larger sample sizes will be useful for further elucidation.

#### Chromosomal evolution as a source of NFAT diversification

Chromosome rearrangement plays a crucial role in the evolution of immune genes^15,49^. The location of each NFAT gene on the vertebrate chromosomes was explored to investigate the influence of chromosomal evolution on NFAT diversification. Similar to the diverse chromosomal locations observed in several immune genes^30^, we identified many locations for all NFATs (76, 48, 51, 23, and 35 for NFATc1–c4, 5, respectively; Table S4), suggesting the frequent incidence of chromosomal rearrangements. Chromosomal rearrangement is an evolutionary process that affects gene duplication^50,51^. Consistently, we found a positive correlation between chromosome and duplication ratios in NFATs (*R*^2^ = 0.77, *p* = 0.01, Fig. 2B), suggesting that the NFAT gene locations affected their gene duplication events in vertebrates.

**Figure 3:**
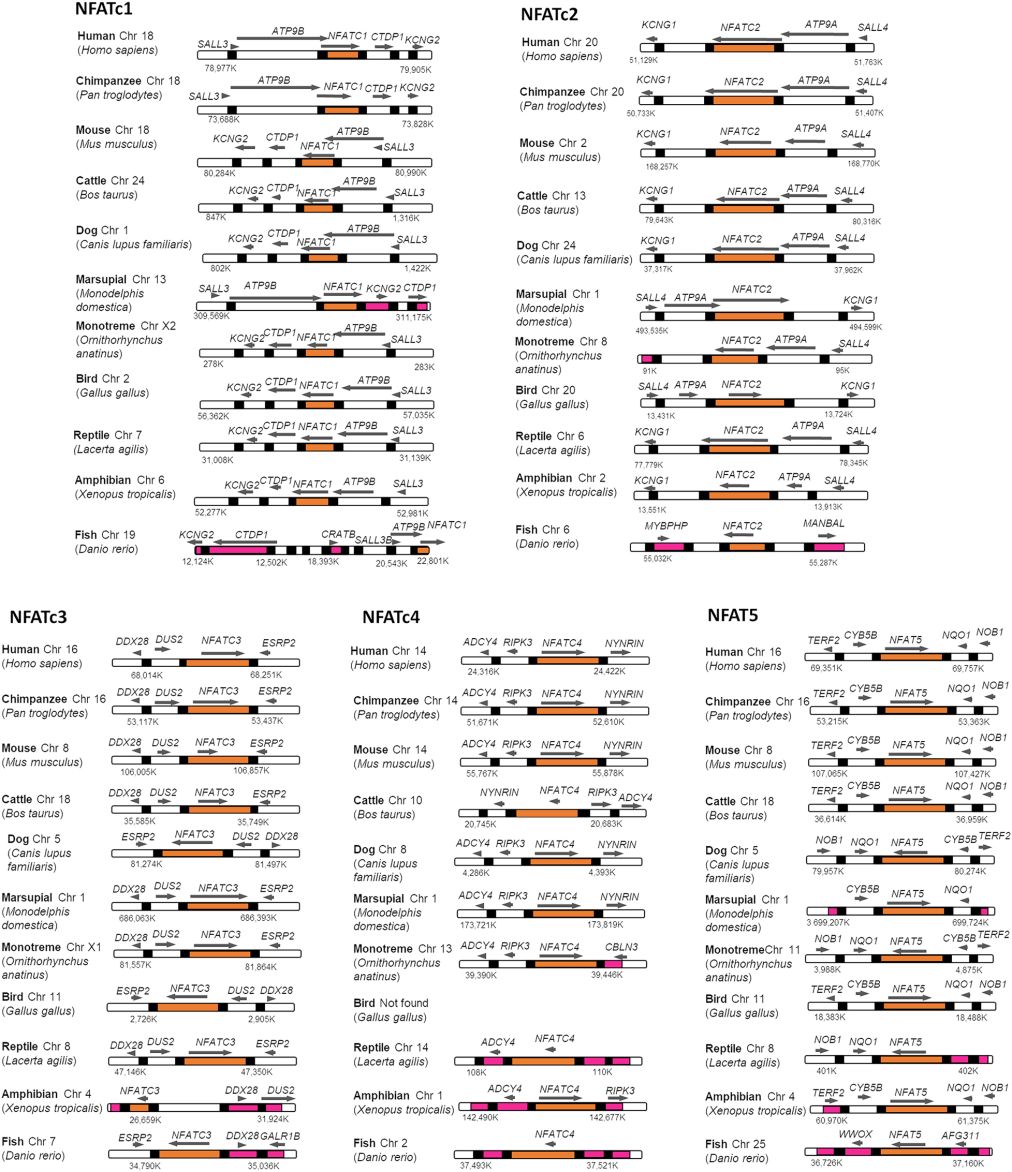
Multispecies comparison of syntenic region of the NFAT genes along the chromosomes. Black rectangle represents the start and/or end of each gene. The NFAT gene region and the evolutionary breaking points are indicated by orange and pink, respectively. The arrow indicates the directionality of the gene region (5′–3′).

Consistent organization of immune-related genes have been reported in relation to coordinated expression patterns, facilitated functional interactions, or maintained epigenetic marks^52^. Therefore, we explored the organization of NFATs and their neighboring genes in the chromosomes of the representative vertebrate species. Despite the diverse chromosome locations (Table S4), strikingly conserved organization was observed among the contiguous genes located near NFATs in the vertebrate taxa (Fig. 3), suggesting the inheritance of NFATs with their neighboring genes as a unit. As reported in other immune-related genes^15^, the chromosomal conformation around NFATs might overcome several chromosomal rearrangements during vertebrate evolutionary history. As previously proposed, we also defined 32 evolutionary breaking points (EBPs) displaying a disrupted order between the regions of NFAT and their neighboring genes in comparison with that of a reference species, alongside the synteny block regions^53,54^. Fish showed recurrent EBPs for all NFATs, probably caused by the whole-genome duplication event proposed for their immune-related genes^30,55^. Additional EBPs were observed in marsupial synteny block regions for NFATc1, monotremes for NFATc2, amphibians for NFATc3, and amphibians and reptiles for NFATc4. Upon extrapolation of paleontological evidence^21^, our findings may be associated with the diversification of vertebrates. The major chromosomal rearrangements in fish occurred during the middle Paleozoic, around 416 Mya, during fish diversification. Then, rearrangements in the clade containing NFATc3 and NFATc4 arose during the late Paleozoic, around 330 Mya, in the reptile and amphibian diversification. Finally, rearrangements in the clade containing NFATc1 and NFATc2 occurred throughout the middle and late Mesozoic, around 95–150 Mya in the mammalian evolution. This chromosomal rearrangement-related evolution hypothesis was consistent with our results, thereby indicating the relationships between NFAT gene duplications and divergence time in vertebrates (Fig. S2). The exact role of chromosome rearrangements should be further elucidated by investigating the differences in rearrangement rates among divergent lineages.

#### A positive selection for all NFATs, except NFATc4

Deleterious mutations are purged due to purifying selection, and conversely, beneficial mutations are fixed in the population^56^. We investigated the role of natural selection on NFATs across vertebrates by evaluating the fixation probability as the ratio of non-synonymous (N: altering amino acid sequences) and synonymous (S: no amino acid change) substitutions (*w* = dN/dS).

Immune genes are subject to selective pressure to resist pathogenic attacks^57^ and are frequently associated with their adaptive evolution^58^. Positive selection was found for NFATc1-c3 and NFAT5 (Table 1), suggesting the incorporation of structural changes in vertebrates. Consistently, a weak but significant correlation between the chromosome ratio and fixation probability was observed in positively selected NFATs (*R*^2^ = 0.44, *p* = 0.04, Fig. 2C). Genes transported to new chromosomal locations frequently undergo positive selection^59^, hence, chromosomal rearrangements, at least in NFATc1-c3 and NFAT5, might play a role in the fixation of structural changes among vertebrates, as observed in other taxa^58,60^.

**Table 1:** Fixation probability of NFATs across vertebrates.

	Mean	SEM	*p*-value
NFATc1	1.0712	0.049	0.0012
NFATc2	1.1254	0.059	0.0053
NFATc3	1.1060	0.027	0.0056
NFATc4	0.8544	0.110	0.0022
NFAT5	1.0936	0.013	0.0010

The fixation probability was evaluated as the ratio (*w*) of nonsynonymous (N) and synonymous (S) substitutions (dN/dS). The data are expressed as the means, standard errors of the mean (SEMs), and the *p*-values obtained from the LRT of each pairwise comparison for each NFAT.

Conversely, a purifying selection was observed for NFATc4 (Table 1). The purifying selection observed in many genes, including immune-related genes^61^, is responsible for sequence conservation and gene function preservation, whereas it may reduce genetic diversity^62^. The conserved structural changes in NFATc4 could be related to the appearance of pathogens affecting various vertebrate species^57,60^, although the loss of gene diversity of NFATc4 across vertebrates might weaken disease resistance^63^.

In conclusion, the strikingly parallel evolutionary history of NFATs across vertebrates suggests a remarkable association between NFAT evolution and immune system development in vertebrates. Our present findings are promising for encouraging additional research on the evolution-related diversification of NFAT function by incorporating novel molecular analysis technologies and increasing data resources.

## Methods

### Data acquisition

Protein and transcript sequences were obtained from National Center for Biotechnology Information (NCBI), Protein Data Bank, UniProt, and Protein Data Bank Japan. Additional sequences for alternative splicing were also obtained from the human transcriptome database (Supplementary file 1). The analysis was performed based on the basic local alignment search tool (BLAST) homology sequences among five major vertebrate taxa (mammals, fish, birds, reptiles, and amphibians, Supplementary files 2–6). The BLASTp algorithm was used to screen proteins using the default settings. All BLAST hits were filtered, and sequences with an e-value < 0.1 and percent identity > 80% were retained. Invertebrate sequences with a percentage identity of > 60% on the domains of the proteins were selected. Candidate sequences were subjected to the tBLASTn algorithm against the database to confirm sequence identity. Transcript data (e-value threshold < 1) were selected using default settings of the NCBI data filter. Short sequences and those with ambiguous names were discarded.

### Phylogenetic analysis

We analyzed the evolution of NFAT by constructing a protein phylogeny using maximum likelihood (ML) and Bayesian approaches. We aligned 3896 protein sequences of 372 species (Table S1) using Multiple Alignment using Fast Fourier Transform (MAFFT) v.7.487^64^ with default parameters under the WAG evolutionary model (ProtTest v.2.4^65^). Samples with large aminoacidic sequences were trimmed for uniform alignment. The Gblocks software^66^ was used to delete highly divergent regions, which were either unambiguously aligned or saturated by multiple substitutions for more than two samples per species. We obtained an average alignment of 1320 amino acids (± 5 amino acids), representing 97% of the total length of the protein. ML was performed using Randomized Axelerated Maximum Likelihood (RAxML) v.8.2.12^67^ with 10,000 bootstrap iterations. A Bayesian search was conducted in MrBayes v.31.2^68^ with 10^6^ generations, and every 2,500 generations were sampled using default priors. Convergence of the run was reached once the likelihood scores formed an asymptote, and the average standard deviation of the split frequencies remained < 0.01. Before convergence, all trees were discarded and the node support was evaluated using a majority rule consensus. We then combined the data after the construction of the phylogeny for each NFAT separately. We used NF-κB samples from humans (NP_001369555.1), rats (XP_038959153.1), fish (QJQ40089.1), mollusks (AKC01669.1), and copepods (AGS12619.1) (Supplementary file 1) as outgroups on the basis of RHD region similarities proposed in evolutionary diversification studies (11). An incongruence length difference test (ILD,^69^) was conducted to evaluate the congruence of tree topologies between ML and Bayesian using tree analysis with new technology (TNT^70^). The ILD test revealed no significant differences in terms of tree topologies (*p* = 0.91); therefore, both (ML and Bayesian trees) were interpreted as unison trees for further discussion.

### Gene duplication events

We calculated the fraction of gene duplications (duplication ratio) by dividing the number of duplicate nodes by the total number of nodes in the phylogenetic trees of the NFATs. We analyzed pairwise orthologous relationships using the default settings of OrthoFinder v.2.3.2^71^. Briefly, orthologs were determined as reciprocal best hits obtained by BLAST in the tree, and gene duplication events were estimated using a duplication-loss-coalescent model^72^. Quartile analysis was employed to explore the duplication ratio variation among vertebrates using custom scripts in Python. We employed an analysis of variance (ANOVA) test in R^73^ to evaluate differences in the fractions of gene duplication among vertebrate taxa. Additionally, we employed a Pearson correlation coefficient (*R*^2^) between the gene duplication fraction and evolutionary divergence time of vertebrates proposed by fossil records^21^ using scikit-learn in Python^74^.

### Alternative splicing

We obtained 459 transcript samples belonging to 236 species from the databases mentioned in Table S2. We used Trinity v.2.12^75^ to assemble the transcript data and estimate isoform abundance using the align_and_estimate_abundance.pl in Trinity. Alternative splicing has been reported in the NFATs of human and mouse species^38^. Further, to estimate splicing isoforms in other vertebrate taxa, we first assembled human and mouse transcripts. With reference to the human-mouse assembly, transcripts from other vertebrates were aligned using local alignment settings in Bowtie2 v.2.4.4^76^ and sorted using Samtools v.1.13^77^.

Sequence similarity (> 95% BLAST homology) among different tissues has been observed in several mammalian species (human, mouse, pig, and cow), hence, one sequence per tissue was selected as a representative to remove tissue transcript-specific bias. The alternative splicing ratio of NFAT was calculated by dividing the number of alternative splicing isoforms by the total number of transcripts. Statistical differences in the splicing ratio among vertebrate taxa were evaluated using ANOVA with Dunnett’s method. A *p*-value < 0.05 was set to indicate the statistical significance.

### Chromosome evolution

We explored NFAT gene locations across the chromosomes of 563 vertebrate samples curated from NCBI excluding the ambiguously named samples. The chromosome ratio was calculated by dividing the number of NFAT gene-containing chromosomes by the total chromosome number for each taxon (Table S3). *R*^2^ between the chromosome and gene duplication ratios was calculated using scikit-learn in Python.

The synteny block within chromosomes was examined in representative vertebrate species, including humans (*Homo* *sapiens*), chimpanzees (*Pan* *troglodytes*), mice (*Mus* *musculus*), cattle (*Bos* *taurus*), dog (*Canis* *lupus* *familiaris*), marsupial (*Monodelphis* *domestica*), monotreme (*Ornithorhynchus* *anatinus*), bird (*Gallus* *gallus*), reptile (*Lacerta* *agilis*), amphibian (*Xenopus* *tropicalis*), and fish (*Danio* *rerio*). We assessed orthologous genes using the Ensembl Compara database^78^ and visualized the synteny using Genomicus v.100.1^79^. EBPs displaying a disrupted order between the region of NFATs and their neighboring genes were identified using the order in humans as reference. However, gene directionality was not considered.

### Natural selection analysis

Genomic NFAT sequences in representative vertebrate species as described above were extracted from the whole genome using the Genome Region Assembly by Baiting (GRAbB) software^80^. Candidate sequences were subjected to the BLASTx algorithm against the database to confirm sequence identity. These sequences were used to evaluate the fixation probability of each NFAT as the ratio (*w*) of nonsynonymous and synonymous substitutions by calculating pairwise comparisons between vertebrate taxa (mammal-reptile, mammal-fish, etc., Supplementary file 1) using a codon substitution model implemented in the codeml package from Phylogenetic Analysis Using Maximum Likelihood (Paml) software v.4^81^. The values of *w* = 1, < 1, and > 1 indicate neutral evolution, purifying selection, and positive selection, respectively. A likelihood-ratio test (LRT) was implemented to estimate the significance in the form of a *p*-value.

To evaluate the contribution of different fractions (gene duplication, alternative splicing, and chromosome rearrangement) in positive selection, we employed linear models in scikit-learn in Python. The significance of the computed partial correlation coefficients was assessed using a bootstrap analysis.

## Supplementary Information

Supplementary Information 1.

Supplementary Information 2.

Supplementary Information 3.

Supplementary Information 4.

Supplementary Information 5.

Supplementary Information 6.

Supplementary Information 7.

## Data Availability

Nuclear factor of activated T cells (NFAT) plays an important role in the immune system by regulating the transcription of multiple cytokines. All study data are included in the article and/or in the supporting information. Analyses were performed using the aforementioned software and custom Python (v.3) scripts, available at https://github.com/maribetg/NFAT-evolution.
