# Supplementary material for: Evolutionary mechanisms underlying the diversification of nuclear factor of activated T cells across vertebrates
Source: Sci Rep. 2023 May 8;13:6468. doi: 10.1038/s41598-023-33751-6 (PMC10167247; doi:10.1038/s41598-023-33751-6)
Supplement: Supplementary file 7 — Supplementary Information 7. [file 41598_2023_33751_MOESM7_ESM.docx]

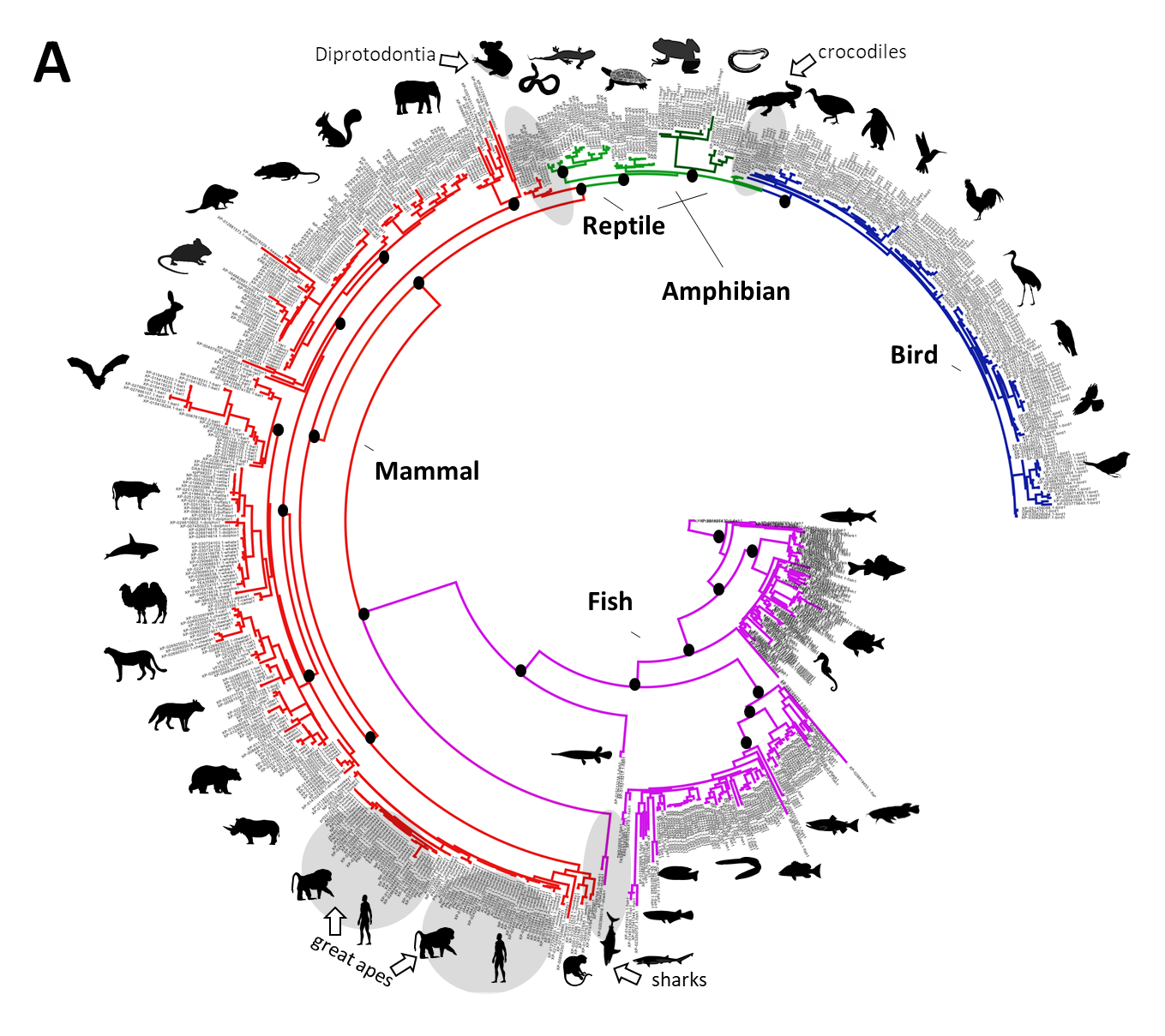


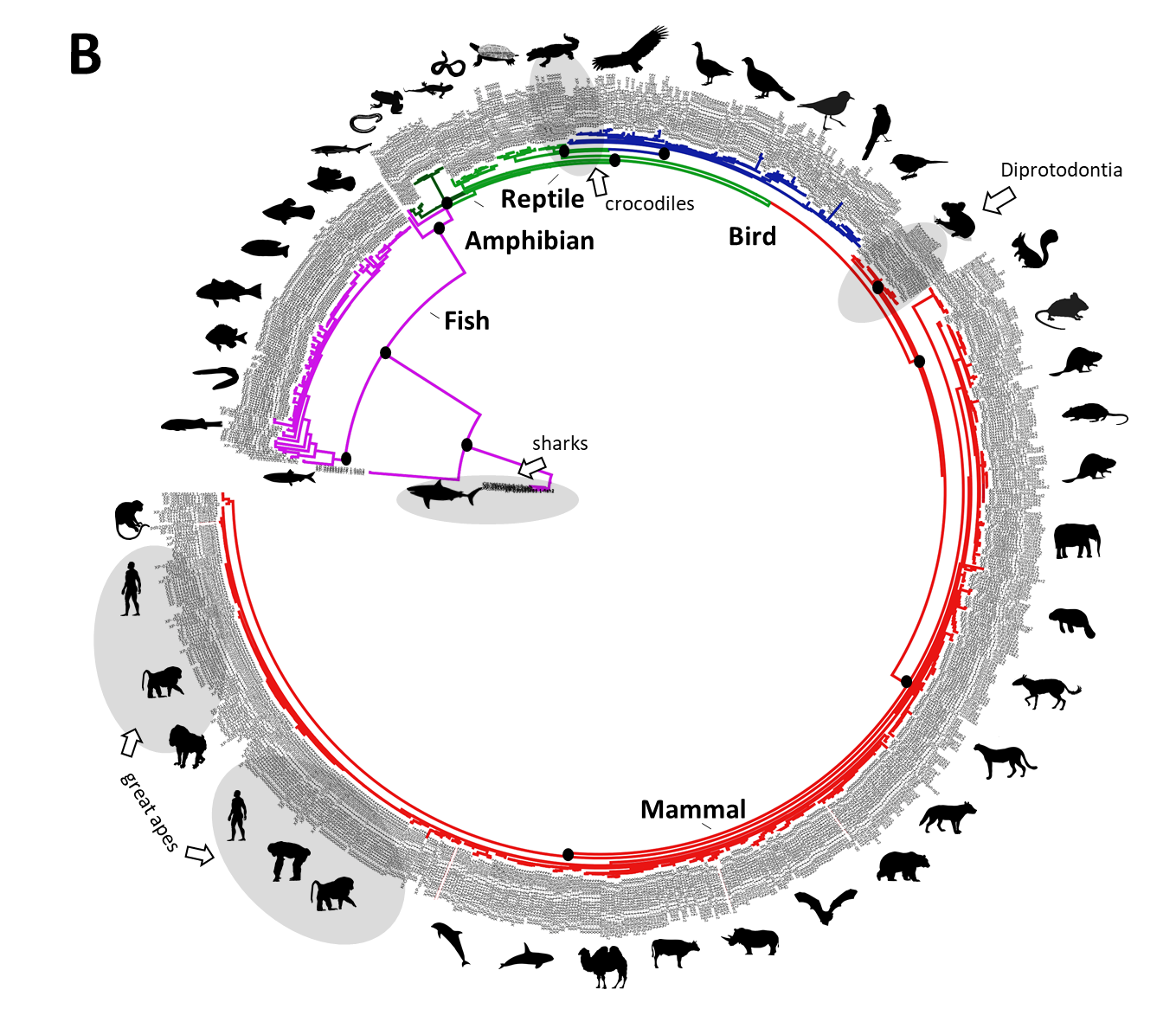


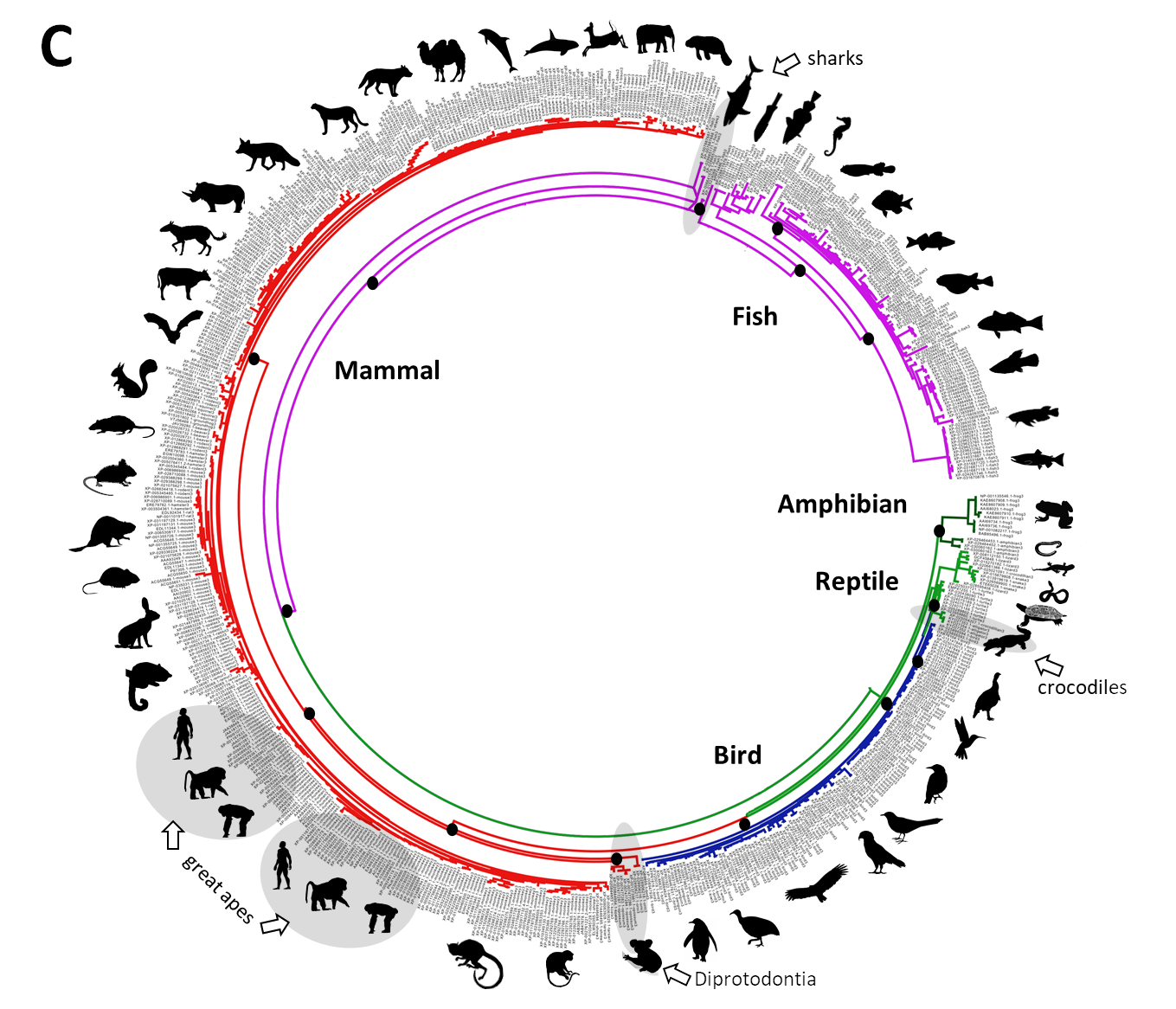


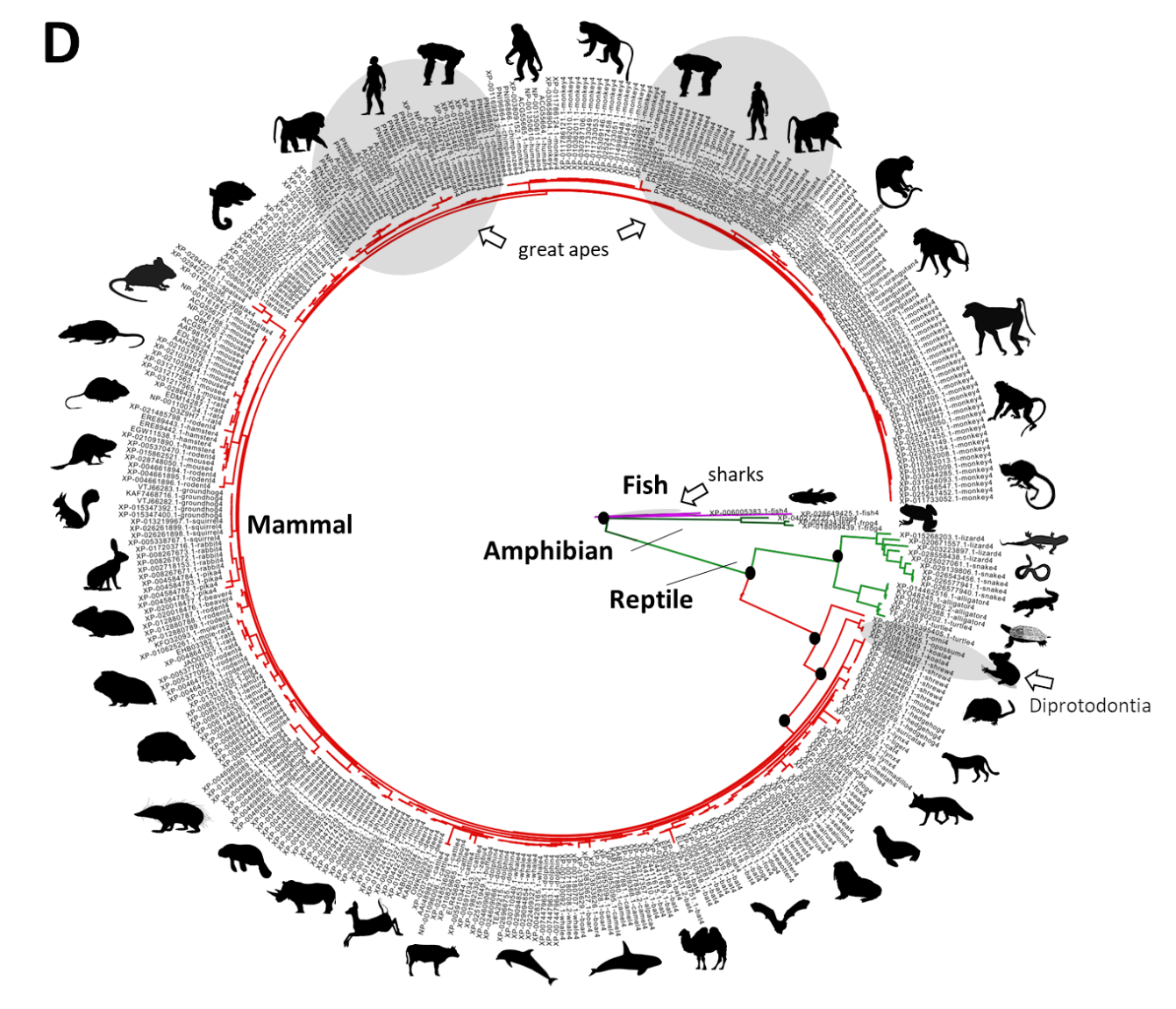


**Fig. S1.** Phylogenetic tree for NFATc1 (A), NFATc2 (B), NFATc3 (C), and NFATc4 (D) across vertebrate taxa. The black dots on the major nodes represent maximum likelihood bootstrap (> 97) and Bayesian posterior probability (> 0.98) support values. Representative species in the major vertebrate branches are illustrated as black silhouette images (http://phylopic.org/). Grey circles show the position of crocodiles, Diprotodontia (koala, opossum, and ornithorhynchus), sharks and great apes. The red, blue, light green, dark green, and purple branches correspond to mammals, birds, reptiles, amphibians, and fish, respectively.


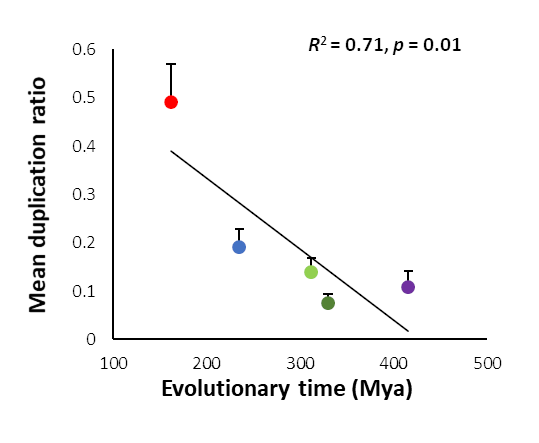


**Fig. S2.** Relationship between duplication events in NFATs and vertebrate evolution. A scatter plot of mean duplication ratio of NFATc1-4 and NFAT5 with standard error of the mean and evolutionary time of vertebrate divergence proposed by fossil records (21) is shown. Red, blue, light green, dark green, and purple dots correspond to mammals, birds, reptiles, amphibians, and fish, respectively. Mya: million years ago.

**Table S1**. Species and protein sequences obtained for NFAT evolution analysis.

| Taxa | Total species | NFATc1 | | NFATc2 | | NFATc3 | | NFATc4 | | NFAT5 | |
| --- | --- | --- | --- | --- | --- | --- | --- | --- | --- | --- | --- |
|  |  | Species | Sequences | Species | Sequences | Species | Sequences | Species | Sequences | Species | Sequences |
| Insect | 27 |  |  |  |  |  |  |  |  | 27 | 37 |
| Mollusca | 1 |  |  |  |  |  |  |  |  | 2 | 2 |
| Star fish | 1 |  |  |  |  |  |  |  |  | 1 | 1 |
| Sea urchin | 1 |  |  |  |  |  |  |  |  | 1 | 1 |
| Tunicate | 1 |  |  |  |  |  |  |  |  | 1 | 1 |
| Lancelet | 1 |  |  |  |  |  |  |  |  | 1 | 1 |
| Fish | 77 | 62 | 209 | 50 | 145 | 61 | 140 | 4 | 8 | 16 | 50 |
| Amphibian | 7 | 4 | 26 | 3 | 22 | 4 | 14 | 3 | 3 | 5 | 6 |
| Reptile | 27 | 16 | 43 | 14 | 51 | 17 | 23 | 14 | 17 | 21 | 59 |
| Bird | 80 | 50 | 155 | 59 | 127 | 65 | 117 | - | - | 52 | 161 |
| Mammal | 149 | 103 | 354 | 118 | 582 | 129 | 391 | 118 | 359 | 126 | 786 |

The numbers of species and protein sequences in vertebrate and invertebrate taxa included in this study are shown. All sequence information is described in S1 Dataset.

**Table S2.** Duplication events in vertebrate NFATs.

| Taxa | NFATc1 | NFATc2 | NFATc3 | NFATc4 | NFAT5 |
| --- | --- | --- | --- | --- | --- |
| Fish | 47 | 8 | 30 | 1 | 5 |
| Amphibian | 18 | 15 | 11 | 1 | 3 |
| Reptile | 32 | 26 | 18 | 1 | 23 |
| Bird | 54 | 23 | 14 | - | 30 |
| Mammal | 226 | 33 | 58 | 10 | 103 |
| **Total** | 377 | 105 | 131 | 13 | 164 |

The duplication events in vertebrate taxa were determined by counting the number of duplication nodes as described in Materials and Methods.

**Table S3.** Alternative splicing isoforms in vertebrate NFAT transcripts.

| Taxa | Number of  total species | Number of  total samples | Number of splicing isoforms | | | | | Splicing ratio |
| --- | --- | --- | --- | --- | --- | --- | --- | --- |
|  |  |  | NFATc1 | NFATc2 | NFATc3 | NFATc4 | NFAT5 |  |
| Fish | 84 | 162 | 1 | 0 | 0 | 0 | 2 | 0.022 ± 0.024** |
| Amphibian | 6 | 18 | 2 | 0 | 1 | 0 | 3 | 0.044 ± 0.041** |
| Reptile | 16 | 31 | 2 | 1 | 1 | 0 | 2 | 0.044 ± 0.097** |
| Bird | 29 | 39 | 13 | 0 | 1 | - | 4 | 0.133 ± 0.086** |
| Mammal | 101 | 209 | 45 | 3 | 9 | 20 | 25 | 0.756 ± 0.124 |
| Total | 236 | 459 | 63 | 4 | 12 | 20 | 36 |  |

Alternative splicing isoforms were identified in vertebrate taxa as described in the Materials and Methods. The numbers of total species, transcript samples, and splicing isoforms in NFATs, and splicing ratio determined by dividing the number of alternative splicing isoforms by the total transcript number are shown. The splicing ratio is expressed as the mean of individual NFAT data ± standard error of the mean. ***p* < 0.01, compared with mammal (Dunnett’s method).

**Table S4**. Chromosomal locations of NFAT genes.

| Taxa | NFATc1 | NFATc2 | NFATc3 | NFATc4 | NFAT5 |
| --- | --- | --- | --- | --- | --- |
| Fish | 1, 2, 3, 4, 5, 6, 7, 8, 9, 10, 11, 12, 13, 14, 16, 17, 18, 19, 20, 21, 22, 23, 25, 27, 31, 34, 36, 37 | 1, 4, 5, 6, 7, 8, 12, 15, 16, 20, 23, 24 | 1, 2, 3, 4, 5, 7, 8, 9, 11, 12, 13, 14, 15, 16, 17, 22, 25, 26 | 2 | 1, 2, 3, 4, 6, 7, 15, 25, 26, 29 |
| Amphibian | 1, 2, 3, 6, 14, 16 | 8, 9, 10, 11 | 4, 5, 7 | 16 | 5 |
| Reptile | 1, 2, 4, 7, 8, 9, 10 | 6, 14 | 8, 9, 12, 14, 15 | 13, 14, 21, Z | 8 |
| Bird | 1, 2, 3, 4, 5, 17, 18 | 3, 10, 13, 16, 17, 19, 20, 22 | 9, 10, 11, 12, 13, W, Z | - | 9, 11, 12, 13, Z |
| Mammal | 1, 2, 3, 4, 5, 6, 7, 8, 9, 10, 11, 12, 13, 14, 15, 16, 17, 18, 19, 20, 21, 22, 24, 28, 30, B4, D3, X2 | 1, 2, 3, 4, 5, 7, 8, 9, 10, 12, 13, 14, 15, 16, 17, 18, 19, 20, 22, 23, 24, A3 | 1, 2, 3, 4, 5, 6, 8, 9, 11, 12, 15, 16, 17, 18, 19, 20, E2, X1 | 1, 2, 3, 5, 6, 7, 8, 9, 10, 11, 12, 13, 14, 15, 17, 22, B3 | 1, 2, 3, 4, 5, 6, 8, 9, 11, 12, 14, 15, 16, 17, 18, 19, 20, E2 |

The chromosome numbers in which NFAT genes are located in vertebrate taxa are shown. All data was obtained from NCBI.
